# The Immune Landscape of Breast Cancer: Strategies for Overcoming Immunotherapy Resistance

**DOI:** 10.3390/cancers13236012

**Published:** 2021-11-29

**Authors:** Kuba Retecki, Milena Seweryn, Agnieszka Graczyk-Jarzynka, Malgorzata Bajor

**Affiliations:** 1 Department of Immunology, Medical University of Warsaw, 02-097 Warsaw, Poland; kuba.retecki@gmail.com (K.R.); milena.seweryn17@gmail.com (M.S.); agraczyk@imdik.pan.pl (A.G.-J.); 2 Laboratory of Immunology, Mossakowski Medical Research Institute, Polish Academy of Sciences, 02-106 Warsaw, Poland; 3 Department of Clinical Immunology, Medical University of Warsaw, 02-006 Warsaw, Poland

**Keywords:** breast cancer, adoptive cell therapy, immune evasion, T cells, CAR-T, tumor microenvironment

## Abstract

**Simple Summary:**

Immunotherapy is a rapidly advancing field in breast cancer treatment, however, it encounters many obstacles that leave open gateways for breast cancer cells to resist novel immunotherapies. It is believed that the tumor microenvironment consisting of cancer, stromal, and immune cells as well as a plethora of tumor-promoting soluble factors, is responsible for the failure of therapeutic strategies in cancer, including breast tumors. Therefore, an in-depth understanding of key barriers to effective immunotherapy, focusing the research efforts on harnessing the power of the immune system, and thus, developing new strategies to overcome the resistance may contribute significantly to increase breast cancer patient survival. In this review, we discuss the latest reports regarding the strategies rendering the immunosuppressive tumor microenvironment more sensitive to immunotherapy in breast cancers, HER2-positive and triple-negative types of breast cancer, which are attractive from an immunotherapeutic point of view.

**Abstract:**

Breast cancer (BC) has traditionally been considered to be not inherently immunogenic and insufficiently represented by immune cell infiltrates. Therefore, for a long time, it was thought that the immunotherapies targeting this type of cancer and its microenvironment were not justified and would not bring benefits for breast cancer patients. Nevertheless, to date, a considerable number of reports have indicated tumor-infiltrating lymphocytes (TILs) as a prognostic and clinically relevant biomarker in breast cancer. A high TILs expression has been demonstrated in primary tumors, of both, HER2-positive BC and triple-negative (TNBC), of patients before treatment, as well as after treatment with adjuvant and neoadjuvant chemotherapy. Another milestone was reached in advanced TNBC immunotherapy with the help of the immune checkpoint inhibitors directed against the PD-L1 molecule. Although those findings, together with the recent developments in chimeric antigen receptor T cell therapies, show immense promise for significant advancements in breast cancer treatments, there are still various obstacles to the optimal activity of immunotherapeutics in BC treatment. Of these, the immunosuppressive tumor microenvironment constitutes a key barrier that greatly hinders the success of immunotherapies in the most aggressive types of breast cancer, HER2-positive and TNBC. Therefore, the improvement of the current and the demand for the development of new immunotherapeutic strategies is strongly warranted.

## 1. Introduction

Recent reports of the International Agency for Research on Cancer indicate that breast cancer is the most frequent malignancy diagnosed worldwide with an estimated 2.3 million new cases and nearly 700,000 deaths in 2020 [1]. From the molecular point of view, tumorigenicity in breast cancers can be attributed to three main signaling pathways mediated by estrogen receptor (ER), progesterone receptor (PR), and human epidermal growth factor receptor 2 (HER2). Hence, based on the expression of these receptors four major molecular types of BC can be distinguished, namely luminal A (high ER expression), luminal B (low ER expression), basal-like/triple-negative (lack ER, PR, and HER2 expression), and HER2 overexpressing subtype. Approximately 70% of breast tumors express ERα. The ERα status is a well-established prognostic factor in BCs, and even if ERα signaling plays a major role in tumor progression, ER-positive cancer generally has a more favorable prognosis than other subtypes [2,3]. A HER2-positive subtype is present in approximately 20% of all BCs. It tends to be more aggressive, characterized by a high proliferation rate, and higher recurrence in comparison to the HER2-negative phenotype [4,5]. ER/PR/HER2-negative subtypes are commonly named triple-negative breast cancer (TNBC) and constitute the most aggressive form of BC. TNBC represents about 10–15% of all breast cancers and is characterized by the poorest prognosis, increased rates of recurrence regardless of the stage of disease and resistance to conventional therapies [6]. According to the Cancer Statistics Review (CSR) presented by National Cancer Institute, the overall five-year survival rate for all breast cancer types regardless of tumor grade is about 90%, however, for metastatic BC it drastically drops to 28%. For TNBC patients the five-year survival rate is 77%, while for patients with distant stages only 12%, and the median overall survival for metastatic TNBC (mTNBC) patients is between 12 and 18 months. Importantly, in mTNBC, the response to chemotherapy is dramatically reduced, leading to a decrease in the median survival time to less than 6 months [7].

Cancer immunotherapy has recently emerged as a forefront antitumor strategy. In general, it can be divided into three branches: passive immunotherapy (e.g., trastuzumab), active immunotherapy (e.g., checkpoint inhibitors), and adoptive immunotherapy (e.g., CAR-T cells). Immunotherapy enables the immune system to harness the body’s own ability to recognize and attack cancer cells. Targeting the biomarkers unique for the tumor cells or the immune checkpoints along with their ligands has revolutionized cancer immunotherapy. For instance, targeting the PD-1/PD-L1 axis with immune checkpoint inhibitors produce a substantial antitumor activity and may provide a long-term survival benefit especially for TNBC patients [8,9]. For PD-L1-positive TNBC patients, treated with atezolizumab plus nab-paclitaxel, the median overall survival was ten months longer compared with chemotherapy alone [10]. In the HER2-directed treatments, the use of the humanized monoclonal antibodies (e.g., trastuzumab, pertuzumab), alone or in combination with chemotherapy results in measurable health outcomes [4]. Median overall survival was calculated as 40.8 months (95% CI 36–48) for patients treated with placebo, trastuzumab, and docetaxel, and for those treated with the pertuzumab, trastuzumab, and docetaxel was assessed to 57.1 months (95% CI 50–72) [11]. Notwithstanding, many patients are exhibiting limited response to these therapies and a high risk of disease progression or relapse. Recently, in hematological malignancies, adoptive cell therapy (ACT) has witnessed a significant breakthrough with the development of chimeric antigen receptors (CAR) technology [12]. However, despite numerous attempts, the CAR-based ACT has still only limited effects in solid tumors. One of the reasons is a hostile, immunosuppressive tumor microenvironment (TME) exerting deleterious effects on trafficking, infiltration, and effector functions of T cells or NK cells, thus constituting a key barrier to effective immunotherapy [13].

In this review, we discuss the landscape of immune system elements in breast cancer, mechanisms adopted by breast tumor cells to escape immunosurveillance, and the most recent immunotherapeutic approaches for BC patients. We also address the issue how to improve and overcome key barriers in breast cancer-directed immunotherapies. We focus on the two subtypes of BC with still poor survival prognosis, HER2-positive and triple-negative breast cancer.

## 2. Immune Landscape of Breast Cancers

**Figure 1:**
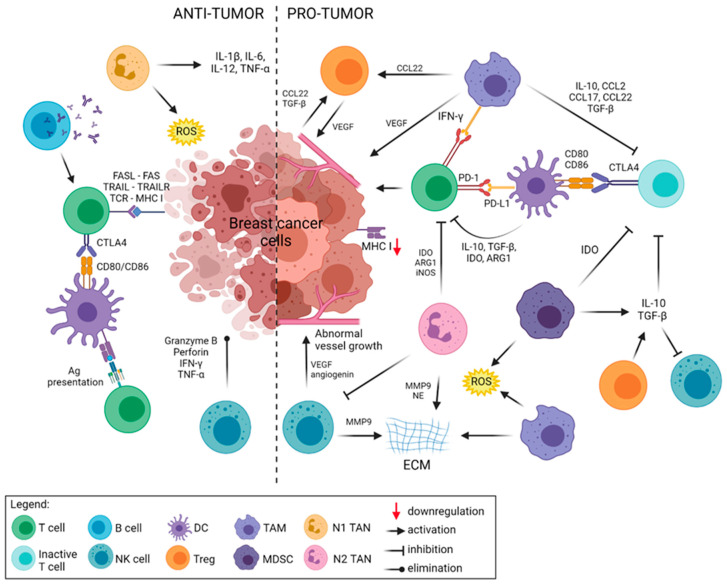
An interplay between breast cancer cells and components of the TME. The anti-tumor and pro-tumor activities of immune cells infiltrating tumor niche. CTLA4—cytotoxic T-lymphocyte associated protein 4, PD-1—programmed death receptor 1, PD-L1—programmed death-ligand 1, VEGF—vascular endothelial growth factor, TNF-α—tumor necrosis factor α, TGF-β—transforming growth factor beta, IFN-γ—interferon gamma, IDO—indoleamine 2,3-dioxygenase, ARG1—arginase 1, iNOS—inducible nitric oxide synthase, MMP9—matrix metalloproteinase 9, NE—neutrophil elastase, ECM—extracellular matrix, ROS—reactive oxygen species, NK cell—natural killer cell, DC—dendritic cell, Treg—T regulatory cell, TAM—tumor-associated macrophage, MDSC—myeloid-derived suppressor cell, TAN—tumor-associated neutrophil. Created with BioRender.com.

The crosstalk between cancer cells and their microenvironment also termed the immune landscape, plays a crucial role in tumor development and progression. Apart from the proliferating tumor cells, the tumor microenvironment is composed of cells that come from the extracellular matrix (ECM), stromal cells (e.g., fibroblasts, mesenchymal cells, endothelial cells, pericytes, adipocytes), along with the innate and adaptive immune system cells (Figure 1). Among them, TME comprises lymphoid lineage cells, such as T cells, natural killer (NK) cells, B cells, and myeloid lineage cells including dendritic cells (DC), neutrophils, and macrophages. The activity of the TME components is also represented by a variety of immunomodulatory factors such as cytokines, chemokines, and growth factors that are examples of major signals secreted by the tumor, stromal, and immune cells responsible for cell-to-cell communication (Table 1).

### 2.1. Components of the Antitumor Immunity

Tumor-infiltrating lymphocytes (TILs) are one of the earliest described populations of immune cells present in the breast cancer tumor microenvironment that were shown to contribute to cancer prognosis and response to therapy. TILs are mainly comprised of cytotoxic (CD8^+^) and helper (CD4^+^) T cells, but also are represented by T regulatory cells, and NK cells [14].

HER2-positive and triple-negative breast cancers are associated with higher TILs levels than ER-positive/HER2-negative breast cancers, indicating that these subtypes are more immunogenic [15]. TILs may be a mirror of a general state of immune activation and the presence of a large number of TILs has been associated with improved clinical outcomes in both HER2-positive BC and TNBC [16]. Moreover, it has been shown that TILs play an essential role in mediating response to chemotherapy and thus, improving clinical outcomes in BC [17]. For instance, the presence of TILs after anthracycline chemotherapy positively correlated with improved overall survival (HR, 0.28; 95% CI, 0.13 to 0.61; *p* = 0.0014) [18]. Likewise, in HER2-positive breast cancer, a positive prognostic association of TILs has been described. The increased TILs numbers have also been shown to be predictive of pathological complete response (pCR) in HER2 positive disease to neoadjuvant therapy with HER2-targeting agents. Thus, the median percentage of TILs infiltration was 20% (4–37%) in TNBC (*n* = 1620 patients), 16% (11–24%) in HER2^+^ (*n* = 2410 patients), and 6% (3–12%) in hormone receptor-positive/HER2^-^ BC (*n* = 929 patients) [15].

In comparison to T cells, the knowledge on the role of B cells in breast cancer is still limited. B cells are known to play an important role in immunological response by producing antibodies and participating in the T cell activation [19]. The role of B cells in tumor immunosurveillance has also been described, where higher densities of tumor-infiltrating B cells (TIL-B) positively correlated with improved clinical outcomes in HER2^+^ BC and TNBC (HR 0.34; 95% CI, 0.12 to 0.95, *p* = 0.03) [20]. Moreover, it has been shown that TIL-B cells were necessary for optimal T cell activation and cellular immunity further supporting their important role in tumor eradication [21].

Natural killer (NK) cells are another population of effector tumor-infiltrating lymphocytes critical to the immunosurveillance and general immune response. NK cells interplay with other immune cells such as dendritic cells, macrophages, T cells, and endothelial cells by producing cytokines and chemokines, and thus, modulate immune responses [22]. NK cells, generally identified as CD3^−^CD56^+^ cells, can be divided into two main subsets, CD56^bright^CD16^low/−^ and CD56^dim^CD16^+^. NK cells can recognize tumor cells in a non-restricted MHC class I manner and eliminate neoplastic cells by releasing cytolytic granules containing perforins and granzymes [23]. On the contrary, NK cells representing a CD56^bright^CD16^+^ subset can facilitate tumor development by releasing the matrix metalloproteinase 9 (MMP9) and secreting the vascular endothelial growth factor (VEGF) and angiogenin [24,25]. In TME, VEGF affects tumor vessel growth and plays modulatory functions by promoting the proliferation of immunosuppressive cells, reducing T cell recruitment, and enhancing T cell exhaustion [26]. Interestingly, the pro-tumor and anti-tumor activity of NK cells depends on BC subtypes. It has been observed that NK cells presence in ER^+^ and HER2^+^ BC patients correlated with a better outcome, whereas in TNBC patients NK cell infiltration in TME corresponded with poor prognosis [27].

Activated dendritic cells (DC) are responsible for the induction of immunological responses, by presenting or cross-presenting the antigens to CD4^+^ and CD8^+^ T cells, which results in the maturation and activation of tumor-specific cytotoxic T lymphocytes (CTLs) that migrate into the tumor niche to recognize and eradicate tumor cells. Tumor-associated DCs displaying immature phenotype can produce proangiogenic factors and facilitate endothelial cell migration contributing to tumor progression [28]. Additionally, tumor-infiltrating DCs show low expression of costimulatory molecules, upregulation of regulatory molecules, and disturbed antigen cross-presentation. In TME, a subgroup of DCs called plasmacytoid dendritic cells (pDC) is associated with a poor clinical outcome, (93% vs. 58% at 60 months), restrained antitumor immune responses and upregulation of regulatory T cells. A higher number of pDCs was found in TNBC when compared to less aggressive, non-metastatic BC subtypes [29].

### 2.2. Tumor Promoting Mechanisms in Breast Cancer

The interplay between tumor cells and the microenvironment, also known as cancer immunoediting, is a complex process consisting of elimination, equilibrium, and escape phases. The elimination phase involves the recognition of tumor-associated “danger signals” by the immune system and the initiation of the inflammatory process, in which innate cells secrete proinflammatory cytokines (IL-12, IFN-γ) to kill cancer cells. Nevertheless, even during continuous eradication of tumor cells, the resistant clonal variants are generated, and this state is defined as an equilibrium phase. Within this phase, if the next cycle of immune response is not able to remove resistant cancer cells the escape phase is reached, which usually gives clinical manifestations. During development and progression tumors achieve the ability to evade the immune system by both intrinsic mechanisms that come from tumor cells themselves and the extrinsic mechanisms which are driven by cells present in TME [30].

#### 2.2.1. Intrinsic Resistance Mechanisms

During tumorigenesis, cancer cells express different antigens on their surface that generally can be divided into two groups, tumor-specific antigens (TSAs) and tumor-associated antigens (TAAs). TSAs are formed as a consequence of somatic mutations that lead to the formation of new amino acid sequences not present in the germline DNA, making them unique for cancer cells. Whereas, TAAs can be presented by HLAs on the surface of both cancer and healthy cells. Thus, TAAs are more challenging targets for therapeutic use due to the lack of specificity or unresponsiveness of the immune system to them [31]. TAAs comprise of differentiation antigens (e.g., melanocyte differentiation antigens), overexpressed cellular antigens (e.g., HER2, MUC-1, CEA), wild-type p53 protein or cancer/testis antigens expressed in germ cells but also activated in cancer cells (e.g., MAGE, NY ESO-1) [32,33]. For example, the melanoma-associated antigen (MAGE-A9) is described to be strongly upregulated in invasive ductal BC and its presence is correlated with unfavorable outcomes (HR 2.377; 95% CI, 1.005 to 5.617; *p* = 0.048) [34]. Overexpression of the HER2 is correlated with poor prognosis, likewise, carcinoembryonic antigen (CEA) overexpression in cancer cells promotes adhesion and metastasis. Thus, TAAs can be targeted for immunotherapy of breast cancer.

Cancer cells are also able to lose antigens to hide from immune surveillance. In order to be recognized by T cell receptors, tumor antigens must be presented in a human leukocyte antigen (HLA)-restricted manner. Impaired HLA-I and HLA-II expressions inhibit the cytotoxicity of T cells and change the antigen presentation by antigen-presenting cells, respectively. Aberrant HLA-G expression by cancer cells inhibits the activity of various immune cells [35]. Thus, decreased expression of MHC I receptors and transporters associated with antigen processing (TAP1 and TAP2) has been correlated with breast cancer grading [36].

Cancer cells to overcome immune surveillance acquired the ability to overexpress inhibitory molecules on the cell surface, including programmed death-ligand 1 and 2 (PD-L1, PD-L2). The activation of the PD-1/PD-L1/PD-L2 pathway induces apoptosis or anergy of T cells, leading to inhibition of anti-tumor activity, induction of immunosuppressive function of T regs, and facilitating tumor growth. Interestingly, a higher PD-L1 expression has been observed in HER2-positive BC and TNBC subtypes rather than in the ER-positive [37]. Similarly to PD-L1, upregulation of the CTLA-4 negatively regulates antitumor T cell-immune response. It was shown that the presence of CTLA-4 on tumor cells is associated with a poor prognosis in breast cancer patients (HR 2.820, *p* = 0.007) [38], however, there are no definite results from the ongoing clinical trials of anti-CTLA-4 antibodies in the TNBC subtype.

In addition, tumor cells can decrease the expression of CD80/CD86 molecules to block the activation of T cells and increase the expression of B7-H1 and B7-H4 molecules to intensify the secretion of IL-10 and induce apoptosis in TILs through upregulation of Fas/FasL signaling in T cells [39].

#### 2.2.2. Tumor-Extrinsic Factors and Mechanisms of Immune Resistance

The extrinsic mechanisms are driven by components present in TME. These are the immunosuppressive cells (e.g., regulatory T cells, macrophages, myeloid-derived suppressor cells, tumor-associated neutrophils) and tumor-derived immunosuppressive factors, such as cytokines (e.g., IL-6, IL-10, TGF-β, TNFα), chemokines and their receptors (e.g., CXCL12, CXCR4), tumor-secreted factors (e.g., VEGF, prostaglandin E2), or enzymes (e.g., IDO, Arg1) that are engaged in suppressing the responses of the immune cells [40].

T regulatory cells (Tregs), apart from controlling autoimmunity and maintaining immunological tolerance, can profoundly affect antitumor responses by releasing the immunosuppressive molecules that impair effector cell cytotoxicity, disruption of metabolism, and modulation of antigen-presenting cells [14]. Tregs are recruited into the TME by cytokines and chemokines released by cancer and immunosuppressive cells. Yan et al. have shown that upregulation of CXCR4 driven by the hypoxic environment impacts the expression of CXCL12 that promotes Tregs recruitment and inhibits the anti-tumor immune response in basal-like breast tumors [41]. A high Foxp3^+^ Tregs infiltration is associated with poor recurrence-free survival in breast cancer patients (combined HR 1.58; 95% CI, 1.03 to 2.44; *p* < 0.0001) [42]. In addition, as a determinant of the prognostic value of cytotoxic TILs in breast cancer, the ratio of total FoxP3^+^ Tregs to CD8^+^ CTLs can be taken into account [43]. Interestingly, in metastatic breast cancer, regulatory T cells can originate from the CD4^+^ T cells as a result of stimulation by the immunosuppressive cytokines such as IL-10 and TGF-β secreted by tumor-evoked B cells [44]. Treg cells presence in TME is associated with an invasive phenotype, worse prognosis, and diminished relapse-free and overall survival of BC patients [45,46].

Tumor-associated macrophages (TAMs) are one of the most abundant and well-studied constitutes of TME [47,48]. Through signals derived from TME, TAMs differentiate into M1-like (anti-tumor) and M2-like (tumor-promoting) phenotypes. In breast cancer, high densities of tumor-associated M2-like macrophages correlated with an increased cancer cell proliferation, metastasis potential and worse prognosis, especially in TNBC (59.0% to 40.4%, *p* = 0.022) [49,50]. M2-like TAMs act in TME by depletion of metabolites (e.g., Arg1), secretion of cytokines and chemokines, and expression of inhibitory molecules (e.g., receptors/ligands for immune checkpoints). Throughout these mechanisms, they altogether inhibit the cytotoxic T cell responses. Tumor-promoting TAMs, similarly to other TME cells, by the production of tumor-promoting factors, including VEGF, various cytokines (IL-10, CCL2, CCL17, CCL22, TGF-β), and matrix-degrading enzymes, facilitate the tumor invasion, angiogenesis, and spread [51]. TAMs can also attract Tregs to suppress antitumor response via secretion of chemokines (e.g., CCL22) [52].

Moreover, the immunosuppressive activity of TAMs is also mediated by IFN-γ -induced expression of PD-L1, which through PD-1 interaction, inhibits the cytotoxic activity of T cells [53]. TAMs can increase the level of the gene encoding the AXL ligand growth arrest-specific protein 6 (GAS6), which contributes to the proliferation and growth of tumor cells, including inflammatory BC [54]. Therefore, regarding the pro-tumorigenic role of TAMs in TME, their targeting might be considered as a therapeutic strategy (reviewed in [55]).

Myeloid-derived suppressor cells (MDSCs) represent a heterogeneous population of immature myeloid cells which promote tumor progression and play a suppressive role in TME. MDSCs presence in BC patients is associated with late-stage disease and poor prognosis [56]. However, the mechanisms underlying MDSCs mediated immunosuppression in breast cancer remain elusive. MDSCs have a high expression of indoleamine 2,3 dioxygenase (IDO), an enzyme that is responsible for the catabolism of tryptophan followed by the production of the kynurenine-based by-products that lead to inhibition of T cell proliferation and induction of T cell apoptosis [57]. Yu et al. have reported that suppression of T cell functions may be regulated by increased activation of STAT3 correlated with activation of the noncanonical NF-κB pathway leading to IDO upregulation [58]. Additionally, the secretion of CXCL2 and CCL22 by the isoform Np63-carrying BC cells has been reported to be associated with MDSC migration to the primary tumor and metastatic sites in TNBC [59]. The proinflammatory cytokines (e.g., IL-10, IL-6, and TGF-β) secreted by MDSCs in TME of BC may facilitate immune suppression [60]. Whereas, IL-33 stimulates the expression and activation of MDSCs and induces the autocrine production of GM-CSF in TME. Moreover, MDSCs demonstrated a strong immunosuppressive activity evoked by the reactive oxygen and nitrogen species (ROS and RNS) production and by attracting other immunosuppressive cells [61].

Neutrophils play a dual role in tumorigenesis by involvement in the progression and suppression of cancer development [62]. An anti-cancer phenotype of neutrophils (N1) is responsible for inflammation processes induced by the release of reactive oxygen species and cytokines (IL-1β, TNF-α, IL-6, and IL-12), however, along with the tumor progression, tumor-associated neutrophils (TANs) acquire a pro-tumorigenic phenotype (N2) and function as suppressors of the immune response. The high frequency of N2-TANs is positively correlated with breast cancer metastasis and resistance to treatment [63]. Factors accumulated in the breast cancer microenvironment remarkably increase TANs persistence, polarization, and expression of pro-inflammatory cytokines [64]. In BC patients, neutrophils have been characterized with an immunosuppressive action towards CD4^+^ and CD8^+^ T lymphocytes impairing their antitumor responses [63]. For instance, TGF-β in TME promotes the formation of pro-tumorigenic neutrophils subtype that is characterized by increased production of nitric oxide synthase (iNOS) and arginase resulting in the suppression of T cell function [65,66]. Moreover, matrix metalloproteinase 9 (MMP9) and neutrophil elastase (NE) secrete by TANs, through the degradation of extracellular matrix (ECM) components, contribute to VEGF release, which in turn promotes tumor vascularization and invasion [67,68,69]. TANs inhibit NK-mediated clearance following the promotion of the metastatic spreading in BC [70], although, in lung cancer, the opposite phenomenon has been observed [71]. The neutrophil to lymphocyte ratio (NLR) is one of the indicators of tumor progression and metastasis. In patients with ER-negative and HER2-negative BC subtypes, a higher NLR correlates with worse prognoses and increased mortality (HR 2.56, 95% CI, 1.96 to 3.35; *p* < 0.001) [72,73].

#### 2.2.3. Environmental Factors Attributed to Cancer Development

It is worth underlining that there are also more general environmental factors that affect the efficacy of the immune response in a variety of cancers—such as age, lifestyle factors, and metabolic disorders. Many studies focused on the direct linking of these factors with the expression of the immune checkpoints and were reviewed in [74]. Aging for instance is associated with the immuno-senescence phenomenon that reduces effector cell proliferative and cytotoxic potential and is generally associated with the elevated levels of checkpoint markers proteins such as PD-1. Unfortunately, while anti-PD-1 therapy improved the survival rate of patients with age < 75 years old, it did not benefit patients with age above 75 years old. This result might be to some extent explained by the immuno-senescence but also raised a question if the activity of T cells should not be tested prior to assigning checkpoint immunotherapy in elderly patients [75]. While the impact of obesity in BC patients remains complex and unresolved [76], the dietary restriction schedules gain some attention as they can switch the metabolic reprogramming within cancer cells that can boost the outcome of the immunotherapy. It was shown that ketogenic stress or AMPK agonists acting via AMPK pathway decrease PD-L1 abundance, enhance antigen presentation and in the end increase the efficacy of anti-CTLA-4 immunotherapy [77]. It was also shown that a ketogenic diet induces changes in the composition of microbiota that result in an increase of 3-hydroxybutyrate (3HB) in serum. 3HB in turn was shown to prevent upregulation of PD-L1 on myeloid cells and, thus, improve the effects of PD-1 blockade [78]. In breast cancer patients enrolled in the IRCT20171105037259N2 clinical trial, chemotherapy was combined with a standard or ketogenic diet. The combination of chemo- and keto-dietary treatment resulted in a decrease of TNFα, an increase of IL-10, lower serum insulin in comparison to the control group, and a significant decrease in tumor burden [79].

## 3. Immunotherapeutic Strategies in Breast Cancer

**Figure 2:**
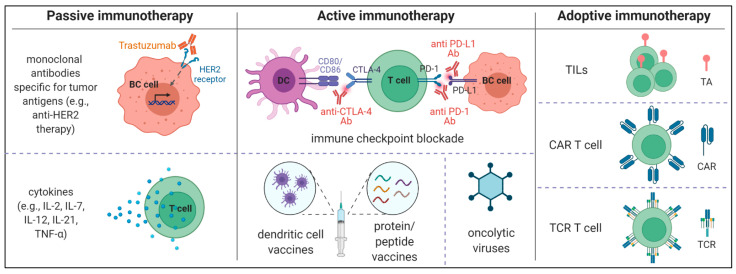
Immunotherapeutic strategies in breast cancer. BC cell—breast cancer cell, IL—interleukin, TNF-α—tumor necrosis factor-alpha, DC—dendritic cell, Ab—antibody, TILs—tumor-infiltrating lymphocytes, TA—tumor antigen, CAR—chimeric antigen receptor, TCR—T cell receptor. Created with BioRender.com.

The immunotherapeutic strategies can be classified into three types: passive, active, and adoptive immunotherapies (Figure 2). The passive immune strategy is related to the use of monoclonal antibodies, for instance, in breast cancer directed towards HER2, such as trastuzumab or pertuzumab. Active immunotherapy, which involves setting an immune response to kill cancer cells, includes primarily the use of checkpoint inhibitors [80]. In breast cancer, these therapies are currently used with considerable success, however, still, many patients acquire resistance to them. Namely, during the analysis of 27 studies including 1746 patients, an objective response was observed in 35% (95% CI = 19–50%) of patients who received the first-line immunotherapy and 22% (95% CI = 12–35%) of patients treated with second-line immunotherapy [81]. Therefore it is important to improve them and concomitantly search for new therapeutic solutions. Recently, intensively investigated adoptive therapies are the hope for patients to win the fight against breast cancer. Adoptive cell therapies (ACT) include the adoptive transfer of tumor-infiltrating lymphocytes, dendritic cells, natural killer cells, and T/NK cells genetically modified to express CAR molecules as well as engineered T cell receptors. Here, all three branches of immunotherapy that are used or those that are currently under investigation in breast cancer will be outlined. However, the greatest part of the work will be devoted to ACT, especially CAR-based cell therapies.

### 3.1. Passive Immunotherapy: Monoclonal Antibodies for Breast Cancer Treatment

Trastuzumab, the first HER2-specific monoclonal antibody was approved in 1998 [82]. The mechanistic insight in the trastuzumab mode of action was revealed later on, as it was shown that the binding site of trastuzumab embraces the domain IV on the C-terminus of HER2 which is important for pathological homodimerization of this receptor [83]. Since then, the development of HER2-specific antibodies focused on targeting different HER2 epitopes that, in turn, could affect the cellular signaling pathways in alternative manners. For instance, pertuzumab was designed to impair the HER2-HER3 heterodimerization which leads to the inhibition of the intracellular signaling via PI3K/AKT pathway. Along with the studies pinpointing the ways to overcome the resistance towards currently employed antibodies targeting the HER2 receptor, other strategies are applied to improve the functionality of these antibodies. In the case of margetuximab, a modification in the Fc region was introduced that leads to increased NK cell activation and enhanced induction of antibody-dependent cellular cytotoxicity (ADCC) resulting in slightly better clinical outcome (HR 0.76; 95% CI, 0.59 to 0.98; *p* = 0.033) [84]. Moreover, other approaches based on the combination of unique antibodies and novel drugs have been also developed, such as ado-trastuzumab emtansine (T-DM1) [85] alone or in combination with drugs (e.g., ARX788 [86]), trastuzumab deruxtecan [87], or administration of DS-8201a in patients previously treated with T-DM1 [88]. Additionally, the combinations of antibodies and classical chemotherapeutical agents have been latterly profoundly investigated. For instance, the addition of pertuzumab, to the combination of the trastuzumab and docetaxel, was reported to prolong the long-term survival in patients with previously untreated metastatic HER2-positive BC (HR 0.69, 95% CI, 0.58 to 0.82) [11]. Furthermore, adding tucatinib, a small molecular highly specific HER2 tyrosine kinase inhibitor, to trastuzumab and capecitabine therapy extended the progression-free and overall survival of patients after previous long-lasting treatment (HR for disease progression or death, 0.54; 95% CI, 0.42 to 0.71; *p* < 0.001; HR for death, 0.66; 95% CI, 0.50 to 0.88; *p* = 0.005) [89]. Regrettably, the resistance to these drugs is emerging, thus forcing the expansion of other treatment strategies. The chosen clinical trial results for HER2-targeted therapy are displayed in Table 2.

### 3.2. Active Immunotherapies in Breast Cancer

Active immunotherapies target the immune checkpoints on cancer cells as well as in TME and can alleviate immune exhaustion of effector cells and, thus, improve anti-cancer responses. Immune checkpoint receptors, present on the surface of immune effector cells, mainly T cells and NK cells, cooperate with their ligands expressed on antigen-presenting cells (APCs) or target cells and either provide signals necessary for the full initiation of an immune response or maintain immune tolerance. The tumor cells in the immunosuppressive microenvironment can evade immune control by increasing the expression of inhibitory or decreasing the expression of stimulatory immune checkpoints and by shifting the balance of the ligand-receptor interactions can inhibit the activation of effector cells, ultimately leading to tumor immune escape. Besides the well-studied inhibitory immune checkpoint receptor-ligand pairs—CTLA-4 that competes with CD28 for binding to CD80 and CD86 receptors or PD-1 and PD-L1/PD-L2, there are also pairs such as LAG3—MHC class II/Lectins, TIGIT—CD155/CD112, TIM3—Galectin 9/PtdSer/HMGB1, and VISTA and its ligand VSIG-3. Apart from the immunosuppressive checkpoints, the immune milieu consists of checkpoint receptors that co-stimulate the immune response, such as CD28, GITR, CD27, CD40, OX40, or CD137 and their particular ligands (reviewed in [80]).

Due to the lack of elemental defining receptors (ER, PR, HER2), the triple-negative breast cancer-directed antibodies are forced to target other molecules. Among them, the leading role is assigned to immune checkpoint inhibitors. For instance, in the KEYNOTE-12 trial (NCT01848834), in 27 out of 32 patients with mTNBC the overall response rate to pembrolizumab (an anti-PD-1 monoclonal antibody) treatment was 18.5% with a duration of response from 15 up to 47 weeks, with 1 complete response, 4 partial responses and 7 stable diseases. In turn, in the phase I trial in patients with mTNBC treated with atezolizumab, of 27 patients that were enrolled, 3 had partial remission and 2 had complete remission [90]. Additionally, in the phase I trial (NCT01772004) in patients with advanced BC, in the arm treated with avelumab, 9 patients out of 168 responded to the treatment, 1 person had a complete remission [8,9]. It was shown that pembrolizumab in combination with chemotherapy triggered a 13.6% increase in pathological complete response in early-stage TNBC patients in comparison to placebo (95% CI, 5.4 to 21.8; *p* < 0.001) [91]. Similarly, atezolizumab targeting PD-L1 in combination with nab-paclitaxel, a paclitaxel-albumin conjugate, prolonged the overall survival of the metastatic TNBC patients (HR for progression or death, 0.80; 95% CI, 0.69 to 0.92; *p* = 0.002) [10]. However, not all combinations of drugs and checkpoint inhibitors were proved to be successful. Although the co-administration of pembrolizumab and enobosarm, a selective androgen receptor modulator, in the androgen receptor-positive subtype of TNBC, showed only a modest antitumor response [92], it still warrants further studies exploring the use of the checkpoint inhibitors. Moreover, to increase the expression of the target checkpoints, a broad range of drugs is constantly investigated, including the classical ones. For instance, doxorubicin and cisplatin pretreatment induced the expression of genes involved in PD-1/PD-L1 signaling and T cell cytotoxicity, leading to a stronger response of PD-1 blockade by anti-PD-1 antibodies [93]. Furthermore, sacituzumab govitecan (SG), an antibody-drug conjugate (ADC), consisting of an anti-Trop-2 (trophoblast cell-surface antigen 2) antibody and active metabolite of irinotecan (SN-38), a topoisomerase I inhibitor, exhibited an advantageous response against TNBC in many clinical trials. SN-38 by inhibiting topoisomerase I, stops ligation of the DNA, leading to DNA breakage and subsequent cell death. Trop-2-dependent delivery of SN-38 induces tumor cell death, and as a bystander effect, once released into the tumor microenvironment, it also targets adjacent tumor cells. Moreover, as an antibody-drug conjugate, SN-38 is characterized with less toxicity when compared to administrated alone [94,95,96]. In turn, ladiratuzumab vedotin (LV), an ADC constructed of an anti-LIV-1 humanized antibody that targets the zinc transporter and a monomethyl auristatin E (MMAE), a potent microtubule-disrupting agent, coupled with a proteolytically cleavable linker. The mode of its action is binding to the LIV-1, then internalization, trafficking to the lysosome, and releasing the payload after proteolytic cleavage of the linker, and subsequent disruption of microtubules. LV is currently thoroughly tested in clinical trials in metastatic TNBC (NCT01969643, NCT03310957) and shows encouraging antitumor activity and tolerability [97,98].

Active immunotherapy includes also the use of dendritic cell vaccines as DCs are one of the most potent antigen-presenting cells and play an important role in T cell activation. Thus, in order to activate T cell antitumor activity, DCs can be modified with tumor-associated antigen(s), fused with tumor cells, or can be armed in cytokine adjuvants, such as IL-2 [99,100,101]. The use of DC vaccinations brings various clinical outcomes in BC, importantly, DC vaccine administration given to the patient is a safe method in cancer treatment. For instance, vaccination with autologous HER2-pulsed DCs in HER2^+^ BC patients resulted in partial response or even attaining a stabilization of disease in some patients [102]. To date, in BC, numerous clinical trials have been investigated with the use of differently modified DCs. Although DC vaccines applications have not yet yielded spectacular clinical outcomes in BC, it is worth noting, that they do not induce significant treatment-related toxicity and are effective independently on the route of administration, thus their development alone or in combination with other therapies may be justified.

#### 3.2.1. Combination Therapies in Breast Cancer

It is noteworthy that monotherapy employing monoclonal antibodies encounters a spectrum of mechanisms reducing their effectiveness. Among the strategies proposed to overcome those hostile conditions, the primacy can be ceded to broadly understood combined therapies. For instance, atezolizumab in combination with either T-DM1, or trastuzumab, or pertuzumab activates the adaptive immune response by exerting pressure on the tumor milieu, leading to the reinforcement in PD-L1 expression and CD8^+^ T-cell infiltration increase [103]. Radiolabeled monoclonal antibody 81c6 targeting tenascin C, an extracellular matrix glycoprotein widely expressed in TNBC cells, in combination with immune checkpoint inhibitors is used in TNBC treatment [104]. Interesting results in a mouse model of TNBC were presented when low dose chemotherapy was combined with oncolytic virotherapy (oHSV-1) followed by checkpoint inhibition (anti-CTLA-4 and anti-PD-L1). Chemotherapy and virotherapy increased the infiltration of TILs in otherwise immune deserted tumors and, strikingly, revealed the importance of the infiltrating B cells as the drivers of the antitumor immunity [105].

To support active immunotherapy, attempts have been made to exploit radiotherapy and its local ablative effect connected with the systemic influence on the immune system. For instance, pembrolizumab administration followed by hypofractionated radiotherapy in patients with heavily pretreated metastatic TNBC reached the ORR of 17.6% (3 out of 17 patients) in the phase II trial [106], however, such treatment combination in ER^+^ BC patients showed no objective response [107]. In turn, the administration of TLR7 agonist—Imiquimod, and cyclophosphamide with subsequent irradiation was investigated in patients with BC with metastases to the skin and demonstrated the local skin response rate up to 83%, unfortunately without the systemic remissions (NCT01421017). Currently, there are clinical trials (NCT04837209, NCT04616248, NCT04756505, NCT03464942) established on merging irradiation with small molecular tyrosine kinase inhibitors, CD40 agonists, bifunctional fusion protein targeting TGF-β and PD-L1, immunocytokine NHS-IL-12, and commonly administered atezolizumab.

#### 3.2.2. Prognostic Significance of Immune Cells in Breast Cancer Immunotherapy

It is worth mentioning that the outcome of immunotherapy also relies to great extent on the immune tumor microenvironment, which in turn can be divided into three profiles—immune-desert, immune-excluded with the T cells at the margins of the tumor, and immune-inflamed [108]. In the randomized phase III KEYNOTE-119 trial, response to pembrolizumab was observed when TILs number was equal or above 5% [109]. Similar results were presented in three other clinical trials: KEYNOTE-173, combining pembrolizumab with chemotherapy [110], NeoTRIP trial assessing atezolizumab in combination with nab-paclitaxel [111] and GeparNuevo, exploring the addition of durvalumab to chemotherapy [112]. The breast cancer tumor microenvironment was also subjected to more detailed stratification using different methods of immune-scoring. Klopfenstein et al. performed in silico analysis of breast cancer transcriptomes that could accurately estimate immune cell populations within the tumor (termed: tumor immune contexture) but also associate those distinct immune profiles with the overall patient survival or assess the risk of the relapse [113]. Another data analysis relying on TCGA samples provided an immune cell infiltration (ICI) score, where high ICI correlated with the suppressed immunity and low ICI score suggested immune-activated phenotype and was an indicator of a positive response of the immunotherapeutic approach [114]. In the case of TNBC, a spatially unique tumor microenvironment was distinguished from T cells infiltrated tumors, namely, stroma-restricted tumors. This type of tumor was characterized by the stromal positivity for PD-L1 and the infiltration of FOXP3^+^CD4^+^ T cells, indicating the development of a potentially immunosuppressive stromal microenvironment [115] and partially explaining the worse overall survival of the patients with this type of tumor [116]. Overall, the more inflamed and infiltrated the tumors are, the better response to immunotherapy is predicted.

To summarize, despite the remarkable advances that have been achieved within passive and active immunotherapies, so far the HER2 targeted therapies in combination with other treatments give some promising results for BC patients, whilst, a multitude of patients with TNBC still await to receive a cure for this deadly disease. High hopes are for adoptive immunotherapies that could make great strides in curing breast cancer.

### 3.3. Adoptive Cell Therapies in Breast Cancer

Adoptive cell therapy (ACT) is a strategy that utilizes autologous or allogeneic transfer of tumor-infiltrating lymphocytes, or T cells genetically engineered to express modified T-cell receptors (TCR) or chimeric antigen receptors (CAR). Briefly, adoptive TILs therapy is based on isolation of TILs from tumor environment, ex vivo activation and expansion with the help of high-doses of interleukin-2 (IL-2), and finally infusion back into the patient. The greatest benefit of using TILs is targeting multiple different and not yet known tumor antigens. In 1987, for the first time, it was shown, that the autologous transfer of ex vivo expanded TILs from different murine tumors, resulted in antitumor activity [117]. After achieving a successful application of ACT with TILs in patients with metastatic melanoma [118], cervical cancer [119], or non-small cell lung cancer [120], TILs effectiveness has also been tested for all breast cancer subtypes and concluded as a reasonable option in treating patients with BC [121]. Unluckily, not all TIL-derived T cells are tumor-responsive, some are characterized by a low survival rate and some can be difficult to activate and expand after reinfusion into the patient’s body. To face these challenges another strategy was employed in the ACT, namely, arming the T cells with the synthetic T cell receptors or chimeric antigen receptors that enable them to target specific cancer antigens. In the case of the TCR-based approach, conventional αβTCRs are genetically engineered to recognize cancer-specific epitopes presented by MHC molecules. Rewiring of TCR recognition leads to an increase of T cell affinity towards cancer cells presenting targeted epitope but on the other hand, has also a serious drawback as it has a limited palette of potential targets to be recognized. Nevertheless, ongoing clinical trials are trying to pave the rationale for the use of TCR-based ACT in BC patients. For instance, in ongoing or recently completed clinical trials the efficacy of the intravenous infusions of TCR-modified T cells against antigens such as HER2, NY ESO-1, and MAGE-A3 alone or in combination with anti-PD-1 therapy in BC patients has been evaluated, nevertheless, with no reported interim results yet (NCT03159585, NCT02111850). Recently, also γδT cells have been taken into account as a modality of ACT therapy [122]. The advantage of γδT cells is their ability to recognize and kill malignant cells in an HLA-independent manner. They also mediate antibody-dependent cellular cytotoxicity and similarly to NK cells express activating receptors that bind their ligands expressed on the malignant cells. It has been shown that antigen-stimulated γδT cells enhanced the efficacy of trastuzumab against HER2^+^ BC cell lines in vivo [123]. Additionally, in TNBC patients, the infiltration by γδT cells into the tumor niche was related to improved outcomes [124]. Up to date, there is limited information regarding the use of NK cells in BC immunotherapeutic approaches. The clear advantage of NK cells over αβT cells is the elimination of cancer cells in an MHC-independent and non-tumor antigen-restricted manner that is to some extent recapitulated by γδT cells. However, NK cells’ activity is inhibited by the immunosuppressive TME and also by the ligation of inhibitory receptors with their cognate ligands on tumor cells. Only one clinical trial regarding the transfer of allogeneic NK cells for a small cohort of BC patients after lymphodepleting chemotherapy and radiotherapy was reported but its results were far from satisfactory [125]. A more promising strategy for solid tumor treatment seems to be the use of CAR-modified NK cells The currently registered clinical trials for the ACT including TILs, TCR-T cells and NK cells are briefly displayed in Table 3.

#### 3.3.1. CAR-Based Therapies in Breast Cancer: Successes and Challenges

Adoptive cell transfer with T or NK cells engineered to express chimeric antigen receptors is one of the particularly promising strategies to fight cancer. CAR is a synthetic molecule composed of an extracellular domain (scFv), that allows surface antigen recognition, combined with intracellular signaling domain derived from physiological T cell receptor (CD3ζ chain), and various co-stimulatory domains (e.g., CD28, 4-1BB, ICOS, OX40) (reviewed in [132,133,134,135,136,137]). CARs can recognize a wide range of cell surface antigens, including glycolipids, carbohydrates, and proteins derived from tumors in a non-MHC-restricted manner which helps overcome MHC downregulation as a mechanism of tumor escape. The use of genetically engineered T cells as a bespoke tailored treatment for leukemias and lymphomas paved a new promising strategy to how cancer can be managed [138]. For breast tumors, several clinical trials have been launched to investigate the value of CAR-T cell therapy (Table 4).

For instance, in the case of pleural mesothelioma, metastatic lung cancer, and metastatic breast cancer the encouraging results for patients who had limited treatment options have been presented in a phase I/II clinical trial of CAR therapy. Patients received an intrapleural infusion of CAR-T cells engineered to target a mesothelin, found on the surface of the cancer cells, plus pembrolizumab (Keytruda^®^), a checkpoint inhibitor that blocks PD-1 molecule on T cells. Out of 14 patients that received combination treatment, two patients acquired complete response, five gained partial response and in the case of four, the disease was stabilized (NCT02414269). Additionally, c-Met, an antigen present in 50% of breast tumors, has been selected for CAR-T therapy of metastatic BC. To increase the safety of CAR-T infusion and minimize on-target/off-tumor effects, T cells were transiently modified with c-Met-CAR construct using an mRNA electroporation method. Intratumorally injected mRNA c-Met-CAR-T cells caused only mild adverse effects but evoked a potent inflammatory response, followed by tumor necrosis and loss of c-Met immunoreactivity as assessed by immunohistochemistry [141]. Recently, Klichinski et al. utilized CAR-modified human macrophages (CAR-Mφ) that demonstrated antigen-specific phagocytosis and tumor clearance in vitro. In solid tumor xenograft mouse models with HER2 expression, administration of human CAR-Mφ decreased tumor mass and prolonged overall survival. In humanized mouse models, CAR-Mφ induced a pro-inflammatory TME and enhanced antitumor T cell activity [139].

#### 3.3.2. Limitations of Adoptive Cell Therapies

Adoptive cell therapies, including CAR-T, have widened the possibilities to combat neoplasms to the formerly unimagined extent. Nevertheless, there are still barriers that contribute to the inefficiency of those technologies. Regardless of the price of the personalized immunotherapies in comparison to the classical or biological pharmaceuticals, there are also particular technical boundaries that limit their utilization. The concept of “the living drug” takes into account an expensive, long-lasting process of preparation of personalized tumor-redirected effector cells. In case of the breast cancer, the TME within the tumor mass acts especially unfavorably against the re-introduced “enhanced” effector cells. The synthesis of immunomodulatory agents such as inhibitory cytokines, biochemical reaction products, and secretory receptors, expression of immunological checkpoint molecules, and direct cytotoxic activity of TME versus effector cells create a genuine obstacle course for adoptive therapy agents. Thus, improving current and developing novel strategies to fight against solid cancers with the help of immunotherapy are still needed.

### 3.4. Alternative Treatment Approaches in Breast Cancer

Alternative cancer treatments are also studied as supplementary to immunotherapy. Recently a broad interest in targeting the oxidative tumor microenvironment evolved as a strategy for cancer treatment. First of all, an attempt has been made to reverse the aggressive and metastatic stage of breast cancer with the help of antioxidant compounds and enzymes [144,145]. Second strategy focus on the induction of extremely high levels of oxidative stress that can trigger apoptosis in cancer cells that are metabolically overwhelmed by the excessively produced ROS/RNS [146,147]. Hence, two opposite anticancer strategies interfering with ROS/RNS, either anti-oxidant or pro-oxidant, should be taken into account. Accordingly, the redox modifiers, intended to act alone or in combination with existing immunotherapies, are already considered as a novel class of promising anticancer agents. For instance, resveratrol and curcumin, both antioxidant agents, encapsulated into immunoliposomes carrying trastuzumab, dramatically increased the antitumor therapeutic effect of these compounds in HER2^+^ breast cancer cells [148]. It has been also shown that curcumin diminishes the inhibition of NK cell tumor cytotoxicity mediated by extracellular vesicles isolated from breast tumor cells [149]. Curcumin may also impair tumor growth through the inhibition of immunosuppressive TME cells, specifically by reprogramming of tumor-promoting M2-like Mφ into anti-tumor M1-like [150]. Moreover, in breast cancer models, curcumin decreased the expression of PD-L1 in cancer cells leading to improvement of antitumor efficacy, and thus sensitizing cancer cells to anti-CTLA-4 therapy [151]. The antitumor and immunomodulatory potential of selenium and its derivatives have been studied in terms of supporting immunotherapy. For instance, selenium supplementation induces a Th1 immune response in breast cancer [152], potentiates the antitumor activity of T cells [153,154], reduces the secondary effects associated with current immunotherapeutic approaches in TNBC [152]. Additionally, sodium ascorbate (vitamin C) has been shown to decrease tumor growth in a T-cell-dependent manner, enhance T cell infiltration into the tumor mass, potentiate clinical efficacy of immune checkpoint therapy with anti-PD-1 and anti-CTLA-4 monoclonal antibodies in murine BC models [155]. Recently, auranofin, a thioredoxin reductase inhibitor, has been found to have anticancer potential. The combination of auranofin with anti-PD-L1 antibody synergistically impaired the growth of murine primary TNBC tumors [156]. Up to date, the alternative approaches with the use of redox-related therapies are mainly focused on boosting the immune system, reducing side effects for other treatment e.g., chemotherapeutic interventions, in general, focusing on improving the quality of life. In conclusion, based on promising preclinical results, clinical trials with redox therapy in combination with checkpoint inhibitors, CAR-T cell therapy, monoclonal antibodies should be implemented in the future. Importantly, future studies should also determine the efficacy and toxicity of the different combination strategies in preclinical models in order to improve the cancer treatment and translation from the laboratory bench to the bedside.

## 4. Strategies to Overcome Immunotherapy Resistance of Breast Cancer

**Figure 3:**
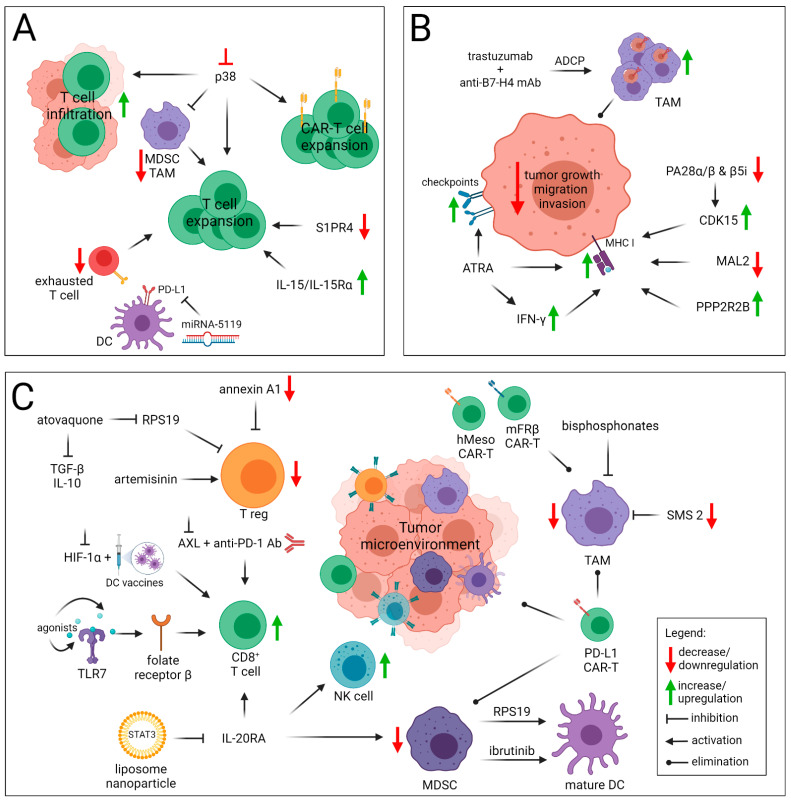
Mechanisms and countermeasures of immunotherapy resistance in breast cancer. Interventions promoting (**A**) expansion and trafficking of T cells within the tumor, (**B**) antigen presentation or target molecule expression, (**C**) limiting the immunosuppressive influence of tumor microenvironment. Depicted actions comprise the molecular modification of the immune cells, administration of stimulatory or inhibitory drugs or combined approaches. The mechanisms presented in the figure are thoroughly described in the text. p38—p38 kinase, S1PR4—sphingosine-1 phosphate receptor 4, CAR—chimeric antigen receptor, IL-15/IL-15Rα—heterodimeric complex of interleukin-15 and interleukin-15 receptor α, PD-1—programed death receptor 1, PD-L1—programed death-ligand 1, MDSC—Myeloid-derived suppressor cells, TAM—tumor-associated macrophages, ATRA—all-trans retinoic acid, B7-H4—coinhibitory molecule, PA28α/β—proteasome activator subunits α and β, β5i—proteasome subunit, CDK15—cyclin-dependent kinase 15, MAL2—M, T-cell differentiation protein 2, PPP2R2B—serine/threonine-protein phosphatase 2A 55 kDa regulatory subunit B, beta isoform, RPS19—ribosomal protein 19, T reg—regulatory T cell, HIF-1α—hypoxia-inducible factor 1α, AXL—AXL receptor tyrosine kinase, TLR7—Toll-like receptor 7, STAT3—signal transducer and activator of transcription 3, IL-20RA—interleukin-20 receptor A, hMeso—human mesothelin, mFRβ—mouse folate receptor β. Created with BioRender.com.

Current immunotherapeutic approaches include efforts to modify the TME within breast tumors thereby making them more responsive to immunotherapy. These strategies include facilitating the trafficking of the expanded cytotoxic cells into the tumor mass, improving the antigen presentation, rewiring hypoxia signaling, or decreasing the inhibitory functions of the components of TME, such as TAMs, regulatory T and B cells, or MDSCs, and also decreasing the activity of inhibitory cytokines. The strategies to enhance the antitumor action, that help to overcome the immunotherapy resistance are summarized in Figure 3.

### 4.1. Enhancing T Cells Priming and Trafficking within the Tumor

The potent antitumor immune response of T cells relies on their proper priming and trafficking into correct localization within the tumor microenvironment. Effector CD8^+^ T cells upon recognition of the antigen and elimination of the target cell bearing that antigen choose one of two pathways. Either they undergo apoptosis or persist as antigen-specific tissue-resident memory CD8^+^ T cells which once re-exposed to the antigen differentiate into effector T cells. During tumorigenesis the amount of exhausted CD8^+^ T cells increases, which are characterized by their decreased cytotoxicity, proliferation, and overexpression of inhibitory molecules (e.g., PD-1). In order to expand T cells, the recruitment of antigen presentation machinery including APCs, activation of the innate immune system, and presence of tumor antigens are required. Therefore, the strategies to boost CD8^+^ T cell expansion and/or activity by targeting mechanisms that inhibit them can be promising in cancer therapy. For example, sphingosine-1-phosphate receptor 4 (S1PR4) promotes mammary tumor progression and limits CD8^+^ T cells survival and proliferation through the regulation of the AKT/PI3K signaling via phosphoinositide-3-kinase adaptor protein 1 (PIK3AP1) as well as leukotriene B4–synthesizing enzyme LTA4H. Olesch et al. have shown that ablation of S1PR4 enhances CD8^+^ T cell proliferation and increases tumor control by the PIK3AP1 and LTA4H regulation [157]. Additionally, IL-15/IL-15Rα, a heterodimeric complex, increases the expansion of CD8^+^ T cells superior to equimolar single-chain IL-15 influencing the T-bet pathway [158]. p38 mitogen-activated protein kinase (p38), a regulator of T cell proliferation and differentiation, contributes to the invasive and metastatic phenotype of TNBC. p38 inhibition improves not only the persistence and tumor infiltration of T cells that results in their boosted antitumor activity but also enhances the functionalities of gene-engineered T cells (with TCR or CAR) [159]. Moreover, the p38 kinase inhibition increases the abundance of cytotoxic CD8^+^ T cells and their tumor infiltration [160]. miRNA-5119 expressing DCs downregulate PD-L1 and not only prevent exhaustion of CD8^+^ T cells but also restore the antitumor activity of exhausted T cells and increase their proliferation [161]. Interestingly, the clinical study with the use of microwave ablation (MWA), a non-invasive, local BC treatment option, has shown that MWA increases the percentage of ICOS-expressing CD4^+^ T cells concomitantly shifting the immunological response to Th1 in HER2^+^ BC and TNBC subtypes [162].

### 4.2. Improving Antigen Presentation

Downregulation or loss of antigen presentation is one of the main tumor escape routes to avoid recognition by immune cells, thus different strategies are used to induce antigen presentation. For instance, all-trans retinoic acid (ATRA), a metabolite of vitamin A1 is currently administered as the cyto-differentiating agent in acute promyelocytic leukemia that activates the antigen-presentation and interferon-related responses in retinoid-sensitive and immune-cold breast cancer tissues. ATRA upregulates the expression of various molecules, including immune checkpoints (e.g., PD-L1). Thus, the use of ATRA in combination with immune checkpoint inhibitors may represent a therapeutic strategy for breast cancer. Bolis et al. have proved that ATRA’s efficiency is higher in the classically immunologically inactive ER-positive BC and lower in TNBC [163]. Another option to enhance antigen presentation and to overcome resistance to HER2 mAb therapies may be activation of antibody-dependent cell phagocytosis (ADCP) by TAMs of HER2^+^ BC patients with poor trastuzumab response. Trastuzumab by inducing overexpression of B7-H4 on TAMs contributes to the poor prognosis, but the concomitant treatment of trastuzumab with the use of the anti-B7-H4 neutralizing antibody markedly increased ADCP of HER2^+^ BC cells and strengthened the response against these cells in vitro and in vivo [164].

Loss or downregulation of MHC class I molecules is widely observed in many cancer types, including BC. The process leading to impairment of antigen presentation, deteriorating the recognition of tumor cells by T cells may be interrupted at every stage. Dhatchinamoorthy et al. have described in detail the mechanisms of loss of MHC I antigen presentation [165], nevertheless, additionally, here we present some newest, interesting reports regarding the issue. The proteasome activator subunits alpha/beta (PA28α/β) proteins that play an essential role in MHC class I processing of certain antigens [166] have been studied in the context of BC therapy. In BC cells, PA28α/β downregulates the expression of serine/threonine-protein kinase (CDK15) which leads to enhanced proliferation, migration, and invasion of tumor cells. Thus, targeting PA28α/β may be the way for inhibiting metastatic breast cancer [167]. Recently, MAL2 has been proposed as a potential target for BC immunotherapy. MAL2 is a raft membrane protein interacting with MHC class I molecules and endosome-associated RAB proteins. In this protein complex, MAL2 is responsible for the late-stage endosome degradation antigen-MHC-I complexes, and thus, reduced antigen presentation on tumor cells. It has been shown that depletion of MAL2, enhanced antigen presentation and significantly improved the cytotoxicity of CD8^+^ T cells against TNBC cell lines in in vitro and in vivo models [168]. Furthermore, the thorough analysis of immunopeptidome, proteome, and transcriptome of the MDA-MB-231 cell line revealed that IFN-γ treatment has an immense impact on the expression of MHC class I-associated molecules such as tapasin [169]. Another recently raised issue was related to the role of PPP2R2B that is expressed abundantly in TNBC patients with longer overall survival. It was shown that the antigen processing and presentation were weakened in the PPP2R2B^low^ tumor cells. Thus, the measures to avoid PPP2R2B loss might be investigated to retain the more benign phenotype of cancer [170].

### 4.3. Overcoming the Immunosuppressive TME

Many different strategies are currently developed to reshape TME by affecting the cytokine signaling, modulating various receptor functions, inhibiting or activating diverse enzymes, targeting hypoxia-driven downstream signaling, changing phenotype and physiology of tumor-infiltrating lymphocytes or tumor-associated macrophages, promoting maturation of immature immunosuppressive myeloid-derived stem cells, reducing Treg number and activity or eliminating TME by the use of CAR-engineered T cells. Recently, Gao et al. have shown that IL-20RA downstream signaling inhibition by a novel liposomal nanoparticle encapsulating STAT3 resulted in CD8^+^ T cell and NK cell recruitment and decreased MDSC proportion in TME [171]. Targeting hypoxia with the use of HIF-1α inhibitor in a mouse model of TNBC, synergized with the DC-based immunotherapy and augmented, both, the cytotoxic and proliferative capacity of cytotoxic T cells [172]. Increased infiltration of T and NK cells accompanied by reduced primary and metastatic tumor burden was also observed when combining AXL inhibitor, with anti-PD-1 therapy in preclinical settings of HER2-positive breast cancer suggesting that alleviating hypoxia can generate a potential therapeutic modality for HER2-positive patients whose tumors exhibit hypoxic signature [173]. Ibrutinib, a Bruton’s tyrosine kinase (BTK) inhibitor, switched the phenotype of MDSC into mature DC and turned the response into Th1 type promoting cytotoxic activity of T cells [174]. Moreover, atovaquone, an antimalarial drug inhibiting the expression of ribosomal protein S19 (RPS19), induced the MDSC maturation, Tregs reduction, and decreased synthesis of TGF-β and IL-10 [175]. Artemisinin, another antiplasmodial drug demonstrated similar activity and evoked an increase of T-bet, IFN-γ, and TNFα levels [176]. Furthermore, administration of TLR7-agonist influencing the folate receptor β-positive TME cells reduced their immunosuppressive function, increased CD8^+^ T cell infiltration, enhanced M1/M2 macrophage ratio, and prompted other profitable anti-tumor responses causing improvement of patients’ overall survival [177]. Inhibition or knock-out of sphingomyelin synthase 2 (SMS2) decreased the formation of M2-like macrophages in vitro [178]. Bisphosphonates reduce the TAM tumor-infiltration rate, which seems to be a remarkable mechanism of their antineoplastic activity [179]. Targeting annexin A1 (ANXA1) leads to Treg function impairment in TNBC [180]. Recently, targeting PD-L1 molecule by CAR-T or NK cells seem to be a promising approach to combat and dismantle the harsh TME. For instance, it has been shown that PD-L1 CAR NK cells not only eradicate cancer cells expressing PD-L1 molecule but also reduce levels of TAMs and other myeloid cells endogenously expressing a high level of PD-L1 in peripheral blood from patients with head and neck cancer [181]. Additionally, PD-L1 CAR-T cells were successfully tested against the tumor microenvironment (stromal ECM and neovasculature) in a syngeneic B16 melanoma model [182]. Additionally, hMeso CAR-T and mFRβ CAR-T showed great efficiency against TAMs. As it was proven, that the expression of FRβ in TAMs is associated with immunosuppressive M2 profile, the elimination of those cells has allowed the infiltration of CD8^+^ T cells and lured pro-inflammatory monocytes to the tumor site. It has been shown that the preconditioning using mFRβ CAR-T enhanced the activity of hMeso CAR-T against cancer cells [183].

### 4.4. Strategies to Overcome CAR-T Resistance

Specificity, precision, and an unimaginable variety of accurately tailored chimeric antigen receptors provide a practically unlimited field for the development of CAR-based ACTs [135]. CAR-T cells, when compared to monoclonal antibodies, demonstrate a substantially higher ability to infiltrate into the tumor site, therefore can be used to support the treatment of antibody-resistant neoplasms [184]. However, CAR-T cells infiltrating the tumor site still encounter obstacles such as troublesome trafficking, immunosuppressive microenvironment, and inhibitory signaling. As CAR-T cells themselves are vulnerable to many immunological and biochemical factors it is essential to improve their performance. The strategies currently being studied include enhancement of the target-directed CAR-T activity. For example, the activity of MUC-1 CAR-T cells toward MUC-1^+^ neoplastic cells was decreased by the secretion of inhibitory cytokines within the TME. In order to enhance MUC1-CAR-T cells were co-expressed with the inverse cytokine receptor linking the IL-4 receptor exodomain to the IL-7 receptor endodomain which converts the suppressive IL-4 signal to one that enhances the antitumor activity of CAR-T cells [185]. Similarly, combining the constitutively active IL-7 receptor with AXL-directed CAR-T also boosted its efficacy in TNBC [186]. Another approach to diminish the impact of inhibitory cytokines was the application of the small-molecular TGF-β-receptor I kinase inhibitor SD-208, to support ROR1-recognizing CAR-T. The combination resulted in prolonged survival, lower PD-1 expression, and constant antitumor function of T cells [187,188]. A similar effect was unleashed by an oncolytic adenovirus rAd.sT that was directed to interrupt TGF-β intracellular signaling, as an augmentation of mesothelin-specific CAR-T. Additionally, the specific killing ability of the anti-HER2 CAR-T cells was reinforced by the addition of IL-21 [189]. Conclusively, such combined treatments displayed a much more impressive antitumor response in comparison to monotherapy [190].

Furthermore, the strategy of interfering with the inhibitory signaling of immune checkpoints is also thoroughly investigated. For instance, anti-HER2 CAR-T augmented with anti-PD-1 monoclonal antibody presented potent cytotoxicity, and thus regressed tumor growth in the trastuzumab-resistant breast cancer cell lines [191]. A deacetylase inhibitor SAHA administered with CAR-T recognizing B7-H3, an immune checkpoint, increased the expression of the target molecule at the transcriptional level and decreased the expression of CTLA-4 and TET2, a methylcytosine hydroxylating enzyme, on effector cells [192].

Likewise, the addition of adjuvant drugs and biochemical agents has become an essential line of aiding CAR-T cells. Therefore, the compounds affecting various intracellular signaling pathways are increasingly administered simultaneously with CAR-T cells. For instance, olaparib, a poly ADP-ribose polymerase (PARP) inhibitor, improved significantly EGFR-specific CAR-T activity in an immunocompetent mouse model of breast cancer. Additionally, it modulates TME, by decreasing the expression of stromal cell-derived factor 1-alpha (SDF1α) and therefore worsening migration of immunosuppressive MDSCs via chemokine receptor CXCR4 [193]. STING agonist, DMXAA, modulates CAR-T cell function such as trafficking, migration, proliferation, but also controls the immunosuppressive agents released by the TME, and thus overall, contributes to the stable regression of tumor after administration of anti-PD1 and anti-GR1 antibodies [194]. In a mouse model of TNBC, it has been shown that the pre-treatment with EGFR-specific CAR-T, induced the development of resistance in tumor cells. This effect could be subsequently reversed by using THZ1, an agent that inhibited the phosphorylation of cyclin-dependent kinase 7 (CDK7)-mediated RNA polymerase II and, therefore, resulted in the diminished expression of the immunosuppressive genes (e.g., *CD274* (PD-L1), *PDCD1LG2* (PD-L2), *IDO1*) [195]. A “headless CAR-T” consisting of intracellular activation domains armed with HER2 or EGFR bispecific antibodies showed great potential in a mono- or sequential killing manner and was able to kill in vitro under hypoxic conditions [196]. Moreover, it was reported that breast cancer stem-like cells succumb to GD2-disialoganglioside-specific CAR-T, which impairs their ability to form lung metastases [197].

Intriguingly, it was observed, that exosomes obtained from mesothelin-specific CAR-T reduce tumor growth inflicting less systemic damage inherently associated with classical CAR-T administration. The mechanism of the cytotoxicity remains unclear, however, a conceivable hypothesis admits the main role to preformed perforin and granzyme B release by CAR-T cell-derived exosomes [198].

Another approach to improve CAR-T effectiveness in hostile TME might be an overexpression or knock-out of the enzymes altering the biochemical capabilities of the T cells leading to priming their proliferation, persistence, survival, and cytotoxic function. For instance, to combat the excess of the reactive oxygen species that are produced in the TME during tumorigenesis, and thus, debilitate immense possibilities of CAR-T, the overexpression of the antioxidant enzymes can be triggered. Ligtenberg et al. has shown that T cells co-expressing CAR receptor and catalase displayed a reduced oxidative state and improved proliferation and cytotoxicity compared with CAR alone [199]. Our recent results indicate that the cytotoxic function of CAR-NK against BC cells, which is suppressed in the oxidative tumor microenvironment, can be enhanced by overexpression of peroxiredoxin-1, a thioredoxin system member scavenging productively ROS [200]. Consequently, the modification of the tumor microenvironment biochemical status using CAR-T armed in the range of various enzymes may turn out to be the way of constructing the super-effective and super-durable killers in the oxidative stress abundant TME.

Furthermore, the replacement of the intracellular domain of the CAR molecule itself may lead to utterly different functionality. An example of such an approach is the use of antitumor-associated calcium signal transducer 2 (Trop-2) specific CAR-T based on the CD27 co-stimulatory intracellular domain. Trop-2 on tumor cells promotes their proliferation and is related to poor prognosis and short overall survival. CD27-based Trop-2 CAR-T cells presented a potent cytotoxic activity, expressed higher levels of IL7-Rα, lower PD-1, and produced more proinflammatory cytokines compared to the CAR-T build of the other tested domains [201]. Moreover, targeting Trop-2 is a promising approach, as the molecule is expressed on many cancer types including TNBC, while in healthy human cells it can be detected only on trophoblast, prostate stem cells, and liver oval cells.

Another tested approach is CAR-NK cell technology that might become a significant branch of adoptive therapies [202,203,204,205,206,207]. Since activation of NK cells does not result in cytokine release syndrome as observed during T cells activation, the harmful consequences of CAR-NK cell treatment can be lower than those of CAR-T cells. Additionally, in opposite to CAR-T therapy, patients undergoing CAR-NK cell treatment are unlikely to suffer from graft-versus-host disease (GVHD). Importantly, CAR-modified NK-92 cells might be an “off-the-shelf” product that could replace the need for the construction of the allogeneic (universal) CAR-T cells [208]. Thus, CAR-NK cell treatment gives hope to overcome the limitations characteristic for CAR-T cells.

## 5. Conclusions

Due to the highly heterogeneous nature of breast cancer, it is difficult to select the one, relevant treatment option which will be precisely tailored for the patient. Although some breast cancer patients benefit from therapies currently in use, still a large group do not respond to treatment or acquire resistance to them. Since HER2-positive BC and TNBC subtypes are characterized as highly immunogenic they also have risen to become targets for immunotherapy which has revolutionized the landscape of cancer treatment. So far, many breakthroughs have been made in immunotherapy of breast cancer, for instance, the use of monoclonal antibodies specifically recognizing antigens on tumor cells (e.g., HER2) or the development of specific antibodies targeting immune checkpoints (e.g., PD-L1, PD-1, CTLA4). Nevertheless, these therapies still need improvement or development of the novel or existing combinatory treatments to be more effective. The success of adoptive cell therapies in hematological malignancies, raised the hopes for breast cancer patients especially, in a view of the use of TILs, TCR-, or CAR-modified T cells. However, it is worth noting these therapies have their advantages and limitations. The main challenges of the adoptive transfer of autologous TILs are the low yield of their expansion and the low affinity of human TCR for tumor antigens. This could be complemented with the use of TCR-engineered therapies that increase antigen specificity but still only when presented in an MHC-dependent manner. Therefore, CAR-based technologies could face these challenges, however, also CAR-T therapies encounter obstacles on the way to be effective. The amelioration of the CAR-T cell efficacy, through precise tailoring of CAR structure, might directly influence the outcome of the antitumor response driven by CAR-T cells in the hostile tumor microenvironment.

## 6. Future Directions

Despite enormous progress in the development of immunotherapy, up to date, none of the current therapies gives spectacular therapeutic effects. Thus, the ongoing efforts focus on the investigation of novel combinations of already known therapeutic regimens [209,210], finding new unique targets [211], or improving the migration and trafficking of effector cells to the tumor site and into the tumor mass, for instance, by genetic rewiring of the integrin and chemokine production [212,213,214]. Moreover, future directions for the BC treatment should rely not only on targeting the cancer cells but also components of the tumor microenvironment. The recruitment and activation of TME components are associated with tumor progression, recurrence, and negative clinical outcomes. Therefore, TME cells, when attracted into the tumor niche, can be reshaped by factors released by cancer cells, and inhibit the antitumor activity of immune cells, supporting tumor growth. Thus, targeting not only cancer cells but concomitantly in the tumor surrounding could be an innovative approach in novel immunotherapeutic regimens.

Notwithstanding, it is important to bear in mind, that enhancing effector cell persistence and intratumoral trafficking, or equipping them with a palette of advantageous modulators of antitumor responses against cancer and TME cells strongly relies on the progress in cell engineering techniques. Indeed, the development of new clinically applicable strategies is strictly intertwined with the progress of preclinical studies in BC research. Thus, each successful implementation of the novel preclinical approach brings us a step closer to composing a weapon capable of annihilating not only the most resistant of breast cancer malignancies but also the residual burden of the disease.

## Figures and Tables

**Table 1:** Immune cells, immunomodulatory factors and their function in the TME.

Role in TME	Cell Type	Immunomodulatory Factors	Function
Anti-tumor	T cell	IL-1, IFN-γ	Tumor antigen recognition, killing tumor cells, promotion of inflammation in TME
	B cell	Antibodies, IL-6, IL-21	Production of antibodies, T cell activation
	NK cell	Granzyme, Perforin, IFN-γ, TNF-α	Activation of immune cells, MHC class I non-restricted recognition of tumor cells, killing tumor cells
	DC	IL-12, CXCL9, CXCL10	Ag presentation to CD4^+^ and CD8^+^ T cells, T cell activation, induction of immunological response
	M1-like Mφ	IL-1β, IL-6, IL-12, CXCL9, CXCL10, IFN-γ, TNF-α	Tumor cell phagocytosis, promotion of immune response, facilitating cancer cell disruption
	N1-TAN	IL-1β, IL-6, IL-12, CXCL9, CXCL10, CXCL11,TNF-α, ROS	Activation of immune cells, killing tumor cells, promotion of inflammation in the tumor microenvironment
Pro-tumor	T cell	IL-4, IL-6, IL-10, IL-13	Inhibition of immune response, activation of immune checkpoints
	Treg	IL-10, TGF-β	Inhibition of immune response, promotion of tumor vascularization, effector cell cytotoxicity impairment, disruption of metabolism, and modulation of antigen-presenting cells
	NK cell (CD56^bright^CD16^+^)	MMP9, VEGF, angiogenin	Increase tumor vascularization, proliferation of immunosuppressive cells, T cell exhaustion, reduction of T cell recruitment
	DC	CXCL8, TNF-α, VEGF, TGF-β	Inhibition of cytotoxic T cells, upregulation of regulatory T cells, increase tumor vascularization
	N2-TAN	CXCL8, IDO, Arg1, iNOS, MMP9, NE, VEGF	Inhibition of T cells and NK cells, ECM degradation, promotion of angiogenesis
	M2-like Mφ	CCL2, CXCL8, CXCL12, IL-10, TGF-β, Arg1, MMP2/9, VEGF, PGE2, ROS	Promotion of tumor vascularization, inhibition of cytotoxic T cells, promotion T cell differentiation into T reg, ECM degradation
	MDSC	IL-10, TGF-β, IDO, Arg1, MMP9, VEGF, ROS	Inactivation of T cells and NK cells, ECM degradation, promotion of angiogenesis, inhibition of T cell proliferation and induction of T cell apoptosis, attracting immunosuppressive cells

**Table 2:** The clinical trial results for HER2-targeted therapy in BC.

Treatment	Additional Treatment	A Phase of the Study	Clinical Trial ID	No. of Patients	Posted Results
Pertuzumab	Trastuzumab, paclitaxel	Phase II	NCT01276041	70	CR = 15, PR = 27, SD = 17, PD = 1
Trastuzumab Emtansine	-	Phase III	NCT01702571	2185	median OS 95% CI 27.2 (25.5 to 28.7)
Trastuzumab emtansine	-	Phase III	NCT01419197	602	6-Month Survival = 90.9 (87.79 to 94.01) 1-Year Survival = 68.6 (59.91 to 77.28) median OS 95% CI NA (13.14 to NA)
Trastuzumab emtansine	-	Phase II	NCT00509769	112	median PFS 95% CI 4.6 (3.9 to 8.6)
Gemcitabine Trastuzumab Pertuzumab	-	Phase I, II	NCT02139358	15	median PFS 95% CI 6.4883 (2.7807 to 9.0372)
DS-8201a	-	Phase II	NCT03248492	253	median DR 95% CI NA (8.3 to NA)
Trastuzumab	-	Phase I, II	NCT01325207	34	CR = 0, PR = 6, SD = 18, PD = 10; median OS 95% CI 8.7 (5.6 to 17.3)

CR—complete response, PR—partial response, SD—stable disease, PD—progressive disease, CI—confidence interval, OS—overall survival, PFS—progression-free survival, DR—duration of response, NA—not enough events to estimate a standard error for the median survival time.

**Table 3:** Overview of the clinical trials in breast cancer with the application of the TILs, TCR-T cells and NK cells.

Technology	Additional Treatment	Subtype of BC	A Phase of the Study	Clinical Trial ID/Reference	No. of Patients	Posted Results
TIL therapy						
TILs	IL-2	BC	Phase I	NCT01462903	20	-
CD3^+^ or CD8^+^ TILs	Aldesleukin Cyclophosphamide Fludarabine Pembrolizumab	Metastatic BC	Phase II	NCT01174121 [126]	93	-
TILs after stem cell transplantation	Aldesleukin Trastuzumab Paclitaxel Surgery	BC	Phase I	NCT00301730	1	-
TILs (LN-145)	-	Metastatic TNBC	Phase II	NCT04111510	10	-
Autologous Lymphoid Effector Cells Specific Against Tumor cells (ALECSAT)	Carboplatin Gemcitabine	TNBC	Phase Ib	NCT04609215	20	-
TCR therapy						
Neoepitopes	Nivolumab IL-2	HER2^+^	Phase I	NCT03970382	148	-
Neoepitopes	Fludarabine Cyclophosphamide Aldesleukin	BC	Phase II	NCT04102436 [127,128,129]	210	-
Neoepitopes	Pembrolizumab Aldesleukin Fludarabine Cyclophosphamide	BC	Phase II	NCT03412877	10	-
NY ESO-1	Cyclophosphamide Fludarabine Aldesleukin	BC	Phase II	NCT01967823 [130]	10	CR = 1, PR = 5
NY ESO-1	Fludarabine Cyclophosphamide	BC	Phase I	NCT02457650	36	-
NY ESO-1	-	BC	Phase I	NCT03159585	6	-
TAA-specific CTLs	-	HER2^+^	Phase II	NCT03093350	10	median PFS = 69.5 days (13 to 72), median OS = 116 days (37 to NA)
MAGE-A3	Aldesleukin Fludarabine Cyclophosphamide	BC	Phase I, II	NCT02111850	21	-
NK cell therapy						
Activated NK cells	-	BC	Phase I, II	NCT03634501	200	-
NK cells (DF1001)	Nivolumab or Nab paclitaxel	HER2^+^	Phase I, II	NCT04143711	220	-
iPSC-derived NK cells (FT500)	Nivolumab Pembrolizumab Atezolizumab Cyclophosphamide Fludarabine IL-2	HER2^+^	Phase I	NCT03841110 NCT04106167	37 76	-
iPSC-derived NK cells (FT516)	Avelumab Cyclophosphamide Fludarabine IL-2	TNBC	Phase I	NCT04551885 [131]	12	-

CR—complete response, PR—partial response, PFS—progression-free survival, OS—overall survival, NA—not enough events to estimate a standard error for the median survival time.

**Table 4:** Overview of the clinical trials in breast cancer with the application of the CAR-based strategies.

Target	CAR Technology	Additional Treatment	Subtype of BC	A Phase of the Study	Clinical Trial ID/References	No. of Patients
HER2, GD2, CD44v6	multi CAR-T	-	HER2^+^	Phase II	NCT04430595	100
CD44v6	single CAR-T	-	BC	Phase II	NCT04427449	100
HER2	HER2 (EQ) BBζ/CD19t +	-	HER2^+^ with brain metastases	Phase I	NCT03696030	39
	dual-switch CAR-T	-	HER2^+^	Phase I	NCT04650451	220
	single CAR-T	oncolytic adenovirus CAdVEC	HER2^+^	Phase I	NCT03740256	45
	single CAR-macrophages	-	HER2^+^	Phase I clinical trial	NCT04660929 [139]	18
HER2, PD-L1	dual CAR-T	-	HER2^+^ with serosal cavity metastases	Early Phase I	NCT04684459	18
MUC1	huMNC2-CAR44 MUC1	-	metastatic BC	Phase I	NCT04020575	69
	single CAR-T	-	TNBC	Phase II	NCT02587689	20
	single CAR-pNK	-	TNBC	Phase II	NCT02839954 [140]	10
TnMUC1	single CAR-T	Cyclophosphamide, Fludarabine	TNBC	Phase I	NCT04025216	112
Mesothelin	single CAR-T	Cyclophosphamide, AP1903	HER2^-^	Phase I	NCT02792114	186
	single CAR-T	Cyclophosphamide or pembrolizumab	BC	Phase II	NCT02414269	113
EpCAM	single CAR-T	-	HER2^+^, TNBC	Phase I	NCT02915445	30
c-Met	mRNA CAR-T	-	TNBC, metastatic BC	Phase I	NCT01837602 [141]	6
Nectin4/FAP	single CAR-T	-	advanced BC	Phase I	NCT03932565 [142,143]	30
CEA	single CAR-T	-	BC	Phase I	NCT02349724	75
	single CAR-T	-	BC	Phase II	NCT04348643	40
ROR1	single CAR-T	-	TNBC	Phase I	NCT02706392	60
NKG2DL	single CAR-Tγδ	-	TNBC	Phase I	NCT04107142	10
CT303-406	single CAR-T	Cyclophosphamide, Fludarabine	HER2^+^	Phase I	NCT04511871	15
PSMA	UniCAR02-T-pPSMA	Cyclophosphamide, Fludarabine	PSMA^+^ BC	Phase I	NCT04633148	35

GD2—disialoganglioside, MUC1—Mucin 1, TnMUC1—Tn glycoform of mucin 1, EpCAM—epithelial cell adhesion molecule, c-Met—tyrosine-protein kinase Met, FAP—fibroblast activation protein, CEA—carcinoembryonic antigen, ROR1—receptor tyrosine kinase-like orphan receptor 1, NKG2DL—natural killer group 2, member D ligand, PSMA—prostate-specific membrane antigen.
